# Alterations of the amygdala in post-COVID olfactory dysfunction

**DOI:** 10.1038/s41598-025-23015-w

**Published:** 2025-10-15

**Authors:** Divesh Thaploo, Lars-Patrick Schmill, Naomi Behrend, Svea Gerlach, Cornelia Fogel, Anne-Kathrin Ruß, Wolfgang Lieb, Stefan Schreiber, Walter Maetzler, Corina Maetzler, Michael Krawczak, Norman Weinert, Thomas Bahmer, Shimita Raquib, Karin Fiedler, Jörg Janne Vehreschild, Olav Jansen, Coralie Mignot, Schekeb Aludin, Martin Laudien

**Affiliations:** 1 Department of Otorhinolaryngology, Smell & Taste Clinic, Universitätsklinikum Carl Gustav Carus, Technische Universität Dresden, Fetscherstrasse 74, 01307 Dresden, Germany; 2 Clinic for Radiology and Neuroradiology, University Hospital Schleswig-Holstein Campus Kiel, Arnold-Heller-Straße 3, 24105 Kiel, Germany; 3 Department of Otorhinolaryngology, Head and Neck Surgery, University Hospital Schleswig-Holstein Campus Kiel and Kiel University, Arnold-Heller-Straße 3, 24105 Kiel, Germany; 4 Institute of Medical Informatics and Statistics, Kiel University, University Hospital Schleswig-Holstein Campus Kiel, Brunswiker Straße 10, 24105 Kiel, Germany; 5 Institute of Epidemiology, Kiel University, University Hospital Schleswig-Holstein Campus Kiel, Niemannsweg 11, 24105 Kiel, Germany; 6 Internal Medicine Department I, University Hospital Schleswig Holstein Campus Kiel, Arnold-Heller-Straße 3, 24105 Kiel, Germany; 7 Neurology Department, University Hospital Schleswig Holstein Campus Kiel, Arnold-Heller-Straße 3, 24105 Kiel, Germany; 8 Department of Medical Informatics, University Medical Center Göttingen, Robert- Koch-Straße 40, 37075 Göttingen, Germany; 9 Airway Research Center North (ARCN), German Center for Lung Research (DZL), Wöhrendamm, Großhansdorf, Germany; 10 Faculty of Medicine, Institute for Digital Medicine and Clinical Data Science, Goethe University, Frankfurt, Frankfurt am Main, Germany; 11 Department I of Internal Medicine, University Hospital of Cologne, Cologne, Germany; 12 German Centre for Infection Research (DZIF), partner site Bonn-Cologne, Cologne, Germany

**Keywords:** Amygdala, Olfactory cortex

## Abstract

Olfactory dysfunction (OD) as a symptom of COVID-19 has received significant attention in research due to its high prevalence. While it is transient in the majority of individuals, post-COVID OD persists in a notable subset of patients even months to years after the acute infection. A deeper understanding of the underlying factors driving this phenomenon is essential. There is increasing evidence for an involvement of the central nervous system in this deficit. The objective of this study was to investigate the structural connectivity and integrity of white matter pathways in brain regions associated with olfactory processing using MRI with diffusion tensor imaging (DTI) in patients with persistent post-COVID OD. The study involved 61 patients, divided into two groups: 31 participants with post-COVID OD (PC-OlfDys) and 30 post-COVID normosmic controls (PC-N). For MRI analyses, a region of interest (ROI)-based approach and voxelwise statistical comparisons between the groups with age as a covariate was used. Fractional anisotropy (FA) in the left amygdala was higher in the PC-OlfDys than in the PC-N group, and radial diffusivity (RD) in the right amygdala was higher in the PC-OlfDys group than in PC-N. The PC-OlfDys group exhibited higher depression and anxiety scores, as measured by the eight-item Patient Health Questionnaire depression scale and the Generalized Anxiety Disorder 7 questionnaire, respectively. This study shows that post-COVID OD is associated with significant changes in the myelination or axonal diameter of olfactory-related brain regions. As the amygdala, putamen and piriform cortex (all involved in olfactory function and emotional well-being) showed associations with depression and anxiety scores, we hypothesise that post-COVID OD and depression and anxiety are interrelated, although the direction of this relationship remains to be elucidated.

## Introduction

The olfactory aspect of COVID-19, often presented as reduced smell sensitivity (hyposmia) or complete loss of smell (anosmia), has received much attention in research^1^. While the acute symptoms of viral infection may subside after a few weeks for many individuals, a significant proportion continues to struggle with a range of persistent health issues including persistent olfactory dysfunction (OD) long after the initial infection has cleared^2,3^. Different terms for these long-term effects of COVID-19 have been established. According to the World Health Organization (WHO), post COVID-19 condition is defined as the continuation or development of new symptoms 3 months after the initial SARS-CoV-2 infection, with these symptoms lasting for at least 2 months with no other explanation^4^. The National Institute for Health and Care Excellence (NICE) differentiates between long COVID, which sums up all symptoms persisting after four weeks past infection, and post-COVID-19 syndrome, defined by persistence over 12 weeks^5^. Understanding the underlying factors driving this phenomenon is crucial to developing effective management strategies and providing appropriate support to those affected.

A recent article outlined the impact these persistent symptoms have on patients who are not hospitalised^6^. Since many of the symptoms of long COVID patients, including fatigue and OD, involve brain function, it is obvious to look for cerebral changes that might explain them. It has been well documented that limbic system alterations are associated with COVID-19^7^. Research has shown reduction in recognition of emotional stimuli after 6–9 months post infection with COVID-19^8^. Apart from limbic system involvement in COVID-19, numerous cerebral alterations have been reported. A recent study revealed gray matter reduction in thalamus, internal capsule, cerebellum, and brainstem as well as a OD^9^. In addition, the olfactory bulb, the first relay centre in the brain for olfactory perception, has often been shown to have a reduced volume in long COVID, although perhaps not as much as in OD of other etiologies^10^. Parosmia, a distorted odor perception, is common after COVID-19, and associated with reduced functional connectivity in key olfactory brain areas such as the primary olfactory cortex (piriform cortex and amygdala), and decision-making areas in the brain (prefrontal cortex)^11^. Given that there is significant cerebral change, it becomes relevant to examine changes in structural connectivity associated with post-COVID OD.

Diffusion tensor imaging (DTI), an advanced MRI technique, offers a unique window into the structural connectivity and microstructural integrity of the brain’s white matter pathways^12^. By mapping the diffusion of water molecules along neural tracts, it is possible to discern subtle alterations in axonal organization, myelination, and overall connectivity, providing valuable insights into the neurological sequelae of COVID-19. In the context of post-COVID OD, diffusion imaging holds promise for uncovering the underlying neural correlates of persistent OD and related symptoms^13,14^. Preliminary findings from diffusion imaging studies have highlighted alterations in white matter integrity and connectivity patterns within olfactory circuits, mainly left amygdala, insula, and parahippocampal gyrus, among individuals with persistent OD post-COVID-19^15^. These alterations may reflect direct viral-induced damage, secondary inflammatory processes, or neuroplastic changes in response to sensory deprivation. Moreover, diffusion imaging offers a non-invasive means of tracking changes in brain structure and connectivity over time, enabling researchers to monitor the progression or resolution of neurobiological abnormalities in long COVID patients. Such longitudinal studies are essential for unravelling the trajectory of neurological recovery and identifying potential predictors of persistent OD and other neurological sequelae. Diffusion metrics, such as fractional anisotropy (FA) reflecting overall white matter integrity, mean diffusivity (MD) indicating tissue damage, and axial (AD) and radial diffusivity (RD) reflecting myelination strength and microstructural integrity, respectively, provide insight into microstructure integrity. However, a disadvantage of many of the studies that have been conducted to date is that they have examined very heterogeneous patient cohorts^1,14^. These studies included patients with mild courses, as well as those hospitalised or in intensive care units. This ultimately distorts the evaluations, making it impossible to determine whether the integrity of the white matter was affected by the SARS-CoV-2 infection or by complications arising from treatment. This calls into question the validity of some previous studies.

Our aim was to investigate if there are differences between two groups of people diagnosed with mild, non-hospitalised cases of COVID-19: a group experiencing ongoing olfactory dysfunction (OD), hereafter referred to as the post-COVID OD group (PC-OlfDys), and a control group consisting of individuals who have recovered, hereafter referred to as the post-COVID normosmic group (PC-N). The main question was how OD affects diffusion metrics and whether these alterations can elucidate the long-term effects of olfactory loss based on a prior SARS-CoV-2 infection rather than complications in the treatment process. The specific hypothesis was that individuals in the PC-OlfDys group would show structural changes in areas previously implicated in reduced functional connectivity.

## Methods

### Patient demographics

This study included 61 participants who had participated in the interdisciplinary COVIDOM study at a university hospital on average 8.56 (± 2.34) months after their infection with SARS-CoV-2. The experiments were conducted according to the Helsinki declaration. COVIDOM aimed to investigate the long-term effects of COVID-19 and was conducted on behalf of the National Pandemic Cohort Network (NAPKON)^16^. The COVIDOM study protocol and procedures were approved by the local ethics committees (Kiel: D537/ 20; Würzburg: 236/20_z; Berlin: according to the coordinating study centre Kiel). Local data protection officers have been informed as required. All participants gave written informed consent prior to their inclusion in NAPKON-POP COVIDOM-study. All participants had a PCR-confirmed infection with SARS-CoV-2 at least 6 months ago and were at least 18 years old at inclusion. Exclusion criteria were claustrophobia, current pregnancy, and medical implants incompatible with MRI. During the initial visit and directly around the MRI assessment, olfactory function was assessed using the Sniffin’ Sticks test (Burghart Messtechnik GmbH, Holm, Germany) as described previously^17^. We used the n-butanol version with the wide step method to examine the threshold (T) and summed it up with the discrimination (D) and identification (I) scores for the Threshold-Discrimination-Identification (TDI) score^18,19^. To classify the outcome clinically, terms ‘normosmia’ and ‘dysomsia’ were used in line with the relevant literature^19^. For the PC-OlfDys group, 31 individuals were recruited, who had persistent OD after their infection with SARS-CoV-2, defined by a TDI score < 31. The PC-N control group consisted of 30 participants who had also been infected with SARS-CoV-2 but were normosmic, defined by a TDI-Score ≥ 31.

### Clinical data

All participants completed extensive questionnaires covering demographic data, smoking status, general medical history, and the course of their acute COVID-19. In the questionnaires, the participants were asked to rate their sense of smell on a ten-point Visual Analogue Scale (VAS) from 0 (= no sense of smell) to 10 (= extremely good sense of smell). They rated it retrospectively for the time before and during their infection with SARS-CoV-2, and at the time of the study, i.e. after their infection. Asked about changes in their olfaction, participants also selected answers to the question ‘Since my infection, I perceive odors…’: ‘…same as before.’, ‘…less intensively than before.’, ‘I don’t know’, and ‘…differently than before (changed quality of smell).’ The last option was interpreted as an indicator of parosmia. To examine neuropsychological changes after the infection, participants were also asked to complete the eight-item Patient Health Questionnaire depression (PHQ-8) and Generalized Anxiety Disorder 7 (GAD-7) questionnaire. The PHQ-8 is a validated screening tool for severity of depressive symptoms with a score ranging from 0 to 24^20^. The cut-off values for mild, moderate, moderately severe and severe depressive symptoms are ≥ 5, 10, 15, and 20. GAD-7 screens for severity of anxiety symptoms, resulting in a score ranging from 0 to 21 with scores of ≥ 5, 10, and 15 as cut-off points for mild, moderate, and severe anxiety^21^. At the onsite visit of the COVIDOM-study, the Montreal Cognitive Assessment test (MoCA) was administered to test for cognitive impairment. It assesses different dimensions of cognitive functions resulting in a total score of up to 30 points. Scores ≥ 26 are considered normal^22^.

### MRI protocol

Diffusion tensor imaging (DTI) was carried out using a 3 Tesla MRI scanner (MAGNETOM Vida; Siemens Healthineers, Erlangen, Germany), employing a 32-channel receiver head coil for image data acquisition. DTI data was acquired using echo planar imaging with GRAPPA having the following parameters: TR (repetition time) = 13,200 ms, TE (echo time) = 101 ms, slice thickness = 1,6.mm, field of view (FoV) = 210 × 210, repetitions = 1, flip angle = 180°. Diffusion scans were obtained at b = 0 and b = 1000 s/mm^2^, utilising 30 diffusion directions. A 3D T1-MPRAGE sequence with iso voxel resolution (1 × 1 × 1) was also acquired with a TR = 2300 ms, TE = 2.27 ms, having a distance factor of 50% and layer oversampling of 45.5%.

### Tract based spatial statistics (TBSS)

Voxelwise statistical analysis of the FA data was carried out using the Tract-Based Spatial Statistics (TBSS)^23^ part of FMRIB software library (FSL)^24^. First, FA images were created by fitting a tensor model to the raw diffusion data using FSL diffusion toolkit (FDT), and then brain-extracted using brain extraction toolbox (BET)^25^. All subjects’ FA data were then aligned into a common space using the nonlinear registration tool FNIRT, which uses a b-spline representation of the registration warp field (Rueckert et al. 1999). Next, the mean FA image was created and thinned to create a mean FA skeleton, which represents the centres of all tracts common to the group. Each subject’s aligned FA data were then projected onto this skeleton, and the resulting data fed into voxelwise cross-subject statistics. The same procedure was repeated to obtain images for mean diffusivity (MD), axial diffusivity (AD), and radial diffusivity (RD). To compare the voxelwise statistics, we employed t-test with age as a variable. Further, ROI based analyses were run as previously described^26^. In addition, a few other ROI involved in olfaction were identified based on literature^13^, namely the amygdala, hippocampus, and thalamus. The reason for using specified ROIs is due to their role in olfaction, which has been well documented^27,28^. Recent articles have also used our previous work, specifically for defining primary olfactory regions^29,30^. We extracted the FA, MD, AD and RD values from each ROI using fslmeants, a FSL based utility tool (fslmeants -i all_FA.nii.gz -o ROI.txt -m ROI.nii.gz) and compared the values between two groups.

### Statistical analysis

Statistical analysis was carried out using GraphPad Prism (version 8 for Windows GraphPad Software, Boston, Massachusetts USA, www.graphpad.com) and IBM SPSS Statistics software (version 29.0.1.1 for Windows, IBM, 2023, www.ibm.com). Mean and standard deviation (± SD) were calculated for metric variables and inter-group comparisons were done using the Mann–Whitney U-test (non-parametric). Independent samples t-test was performed to compare FA, MD, RD and AD values from the ROI between cohorts. Whole-brain TBSS voxelwise analyses were corrected for multiple comparisons using family-wise error (FWE) correction at *p* < 0.05. A two-tailed bivariate using Pearson’s correlation coefficient analysis was further performed between diffusion values and questionnaires of interest, with multiple comparisons adjusted using Bonferroni correction where applicable. P-values < 0.05 were considered statistically significant.

## Results

### Patient demographics

**Fig. 1:**
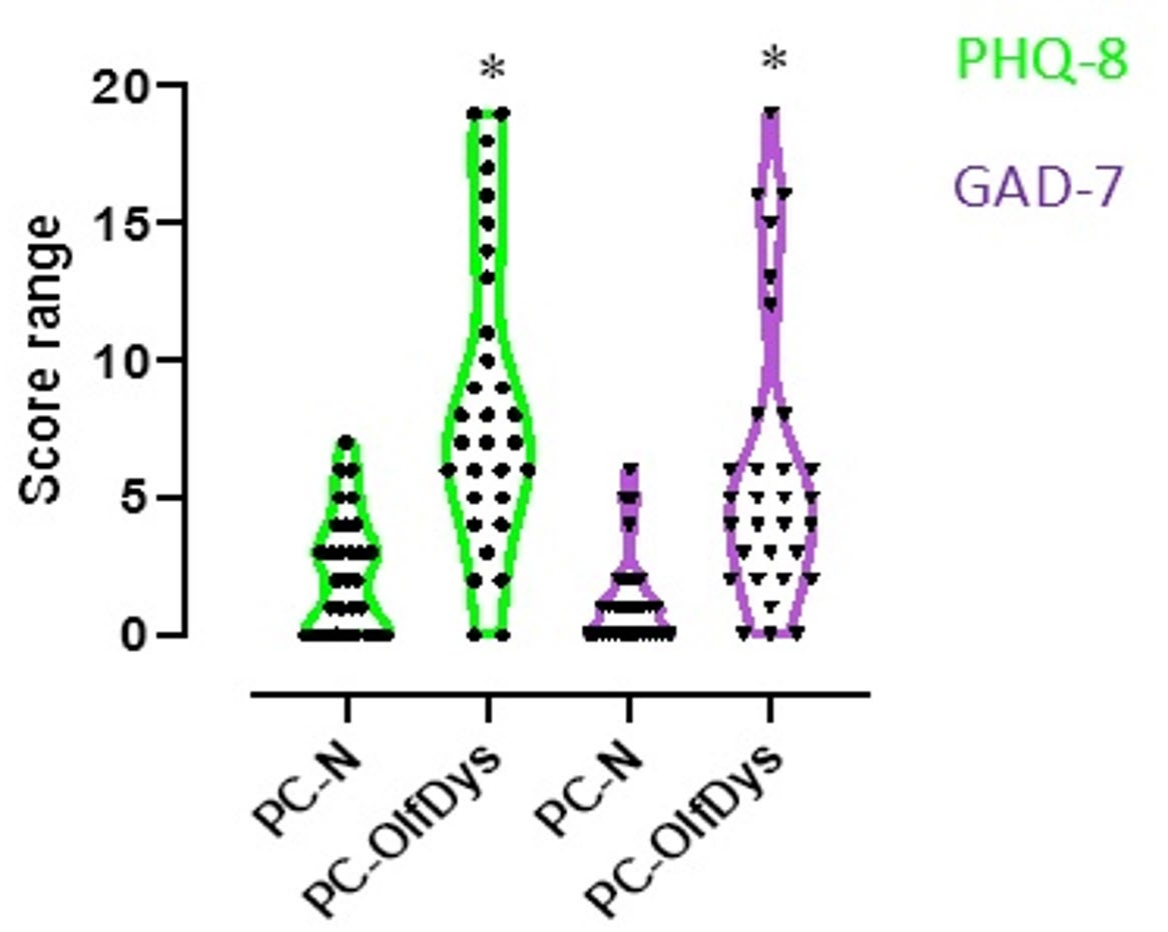
Depicts truncated violin plots of the Patient Health Questionnaire-8 (PHQ-8) and Generalised Anxiety Disorder-7 (GAD-7) scores compared between PC-OlfDys and PC-N group. *p < 0.05.

All participants were examined between April and October 2022 and study demographics are listed in Table 1. The participants in the PC-OlfDys group rated their sense of smell significantly lower during and after infection than the PC-N group (p = 0.007 and < 0.001, respectively). When asked about changes in their smell perception, 10 out of 26 persons (38%) in the PC-OlfDys group and 0 out of 28 (0%) in the PC-N group answered, ‘Since my infection, I perceive odors… differently than before (changed quality of smell)’, indicating parosmia. In the Sniffin’ Sticks test, as intended by the study design, the groups showed a significant difference for all three sub scores and the TDI (*p* < 0.001), with the PC-N group performing better (Table 2). The mean PHQ-8 score was 9 ± 5 in the PC-OlfDys group and 2 ± 2 in the PC-N group (*p* < 0.001). The mean GAD-7 score was 6 ± 5 in the PC-OlfDys group and 1 ± 2 in the PC-N group (*p* < 0.001, Fig. 1). The MoCA was not significantly different between the groups (27 ± 3 in the PC-OlfDys group vs. 27 ± 2 in the PC-N group, *p* = 0.5).

**Table 1:** Demographic characteristics and time since infection with SARS-CoV-2 on the day of MRI scan. w = women, m = men.

Group	Age (years, mean ± SD)	Gender (w/m)	Duration of OD (months, mean ± SD)
PC-OlfDys	44.0 ± 14.2	17/14	18.9 ± 4.4
PC-N	44.4 ± 14.2	17/13	21.9 ± 4.8
p-value	ns	ns	ns

**Table 2:** Clinical measures representing subjective ratings of olfactory function captured using a VAS from 0 to 10. A score of 0 indicates no smell perception, a score of ten an extremely good smell perception. TDI score represents scores obtained from the sniffin’ sticks test. T, threshold; D, discrimination; I, identification score.

Measure	PC-OlfDys (mean ± SD)	PC-*N* (mean ± SD)	*p*-value
Subjective ratings (before infection)	8.1 ± 2.1	8.6 ± 1.3	ns
Subjective ratings (during infection)	1.55 ± 2.5	4.2 ± 4	< 0.05
Subjective ratings (after infection)	3.2 ± 2.2	8.3 ± 1.4	< 0.05
TDI	23.37 ± 3.89	34.42 ± 2.02	< 0.05
T	4.89 ± 2.01	8.48 ± 1.8	< 0.05
D	9.58 ± 1.65	13.17 ± 1.23	< 0.05
I	8.9 ± 2.36	12.77 ± 1.14	< 0.05

### TBSS analysis

Whole-brain TBSS did not show significant group differences after FWE correction (*p* < 0.05) ran using randomise with 5000 permutations.

### ROI analysis

**Fig. 2:**
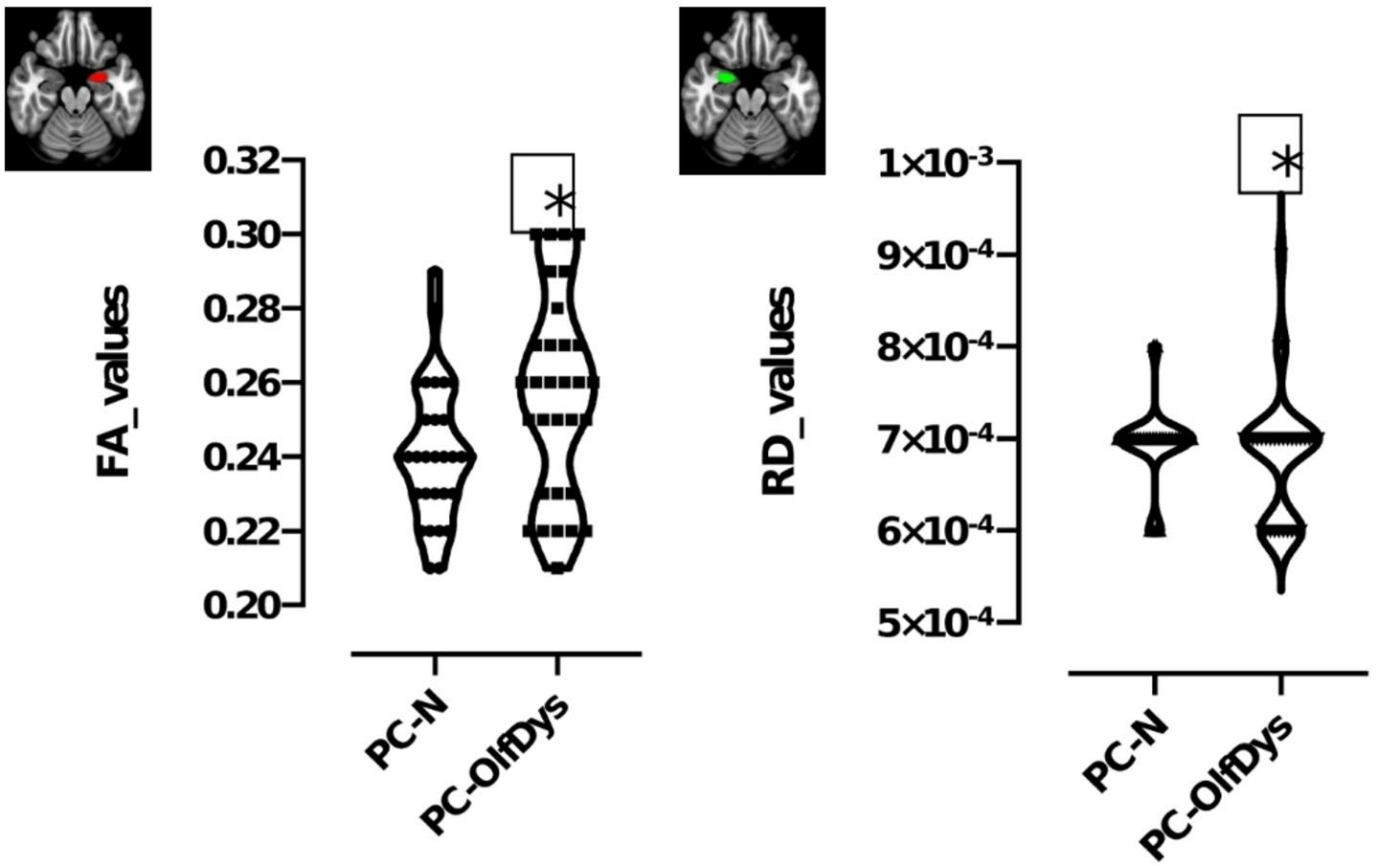
Represents a violin truncated plot of fractional anisotropy (FA) and radial diffusivity (RD) values in left amygdala and right amygdala (marked on a MNI space in red and green, respectively) compared between PC-OlfDys and PC-N group. *p < 0.05.

Independent samples t-test showed that FA values in the left amygdala were significantly higher in PC-OlfDys than in PC-N group (mean ± SD, 0.25 ± 0.02 vs. 0.24 ± 0.01, F = 5.417; *p* = 0.014) and RD values in the right amygdala were higher in PC-OlfDys than in PC-N group (7.1 × 10^− 4^ ± 3.6 × 10^− 4^ vs. 6.8 × 10^− 4^ ± 4.7 × 10^− 4^, F = 1.152, *p* = 0.03) (Fig. 2). All other ROI did not differ significantly in any of the diffusion values.

### Correlations

A bivariate correlation analysis was performed for each group with all diffusion values (FA, MD, RD and AD) from each ROI and PHQ-8, GAD-7, MoCA, and TDI and sub scores.

#### Olfactory function

 In the PC-OlfDys group, the TDI score did not correlate with any of the diffusion values. However, in the anterior piriform cortex, the identification score was negatively correlated with AD values (*r* = − 0.367, *p* = 0.04). In the left amygdala, the discrimination score was negatively correlated with MD (*r* = − 0.389, *p* = 0.03) and RD values (*r* = − 0.491, *p* = 0.005). In the right putamen, the threshold score was negatively correlated with RD values (*r* = 0.375, *p* = 0.03).

Looking at the PC-N group, in the right putamen, the TDI score was positively correlated with MD values (*r* = 0.432, *p* = 0.01). Additionally, in the left amygdala, the identification score was negatively correlated with RD values (*r* = − 0.435, *p* = 0.01).

#### Duration of OD

In the PC-OlfDys group, the duration of OD showed a positive correlation with MD values in the left amygdala (*r* = 0.504, *p* = 0.004) and the left posterior piriform cortex (*r* = 0.385, *p* = 0.03). A positive correlation was also observed between duration of OD and AD values in the left amygdala (*r* = 0.532, *p* = 0.002).

#### PHQ-8

 A negative correlation of PHQ-8 with FA values in the left amygdala (*r* = − 0.414, *p* = 0.02) was seen in the PC-OlfDys group. There was also a negative correlation between PHQ-8 and AD values in the left putamen (*r* = − 0.381, *p* = 0.03). No correlation was seen in the PC-N group.

#### GAD-7

A negative correlation with FA values in left amygdala was seen in the PC-OlfDys group (*r* = − 0.377, *p* = 0.03). No correlation was seen in the PC-N group.

## Discussion

Persistent OD may induce significant alterations in olfactory-related brain regions. Although whole-brain TBSS did not reveal significant between-group differences after FWE correction, the ROI-based approach proved more sensitive in detecting alterations within regions strongly implicated in olfactory processing. This suggests that targeted, hypothesis-driven analyses may provide greater power to uncover subtle yet biologically meaningful effects. Increased FA and RD values within the amygdala region in post-COVID patients with OD, compared to normosmic controls. This may indicate changes of myelination or changes in axonal diameter in the white matter pathways adjacent to the amygdala. Duration of loss was positively correlated with MD and AD values in the left amygdala and posterior piriform cortex. This result suggests that the longer the OD lasts, the more likely it is that these structures will be affected. In addition, people in the PC-OlfDys group had higher scores on the PHQ-8 and GAD-7 questionnaires. It remains to be investigated whether these symptoms are a “behavioural” consequence of persistent OD or whether the symptoms are a consequence of structural changes caused by (post-)COVID (and could be triggered, for example, by an olfactory deficit).

Increased FA in the left amygdala indicates increased myelination, which in turn depicts white matter strength or integrity^31^. This increased myelination can be interpreted as better communication between the amygdala and the ventromedial prefrontal cortex, which, for example, predicts more favourable outcomes in terms of anxiety^32^. This is supported by the present analyses, which show negative correlations between PHQ-8 and GAD-7 scores and FA values in the left amygdala. This essentially means that as FA levels increase, indicating better myelination, anxiety and depression decrease, mediated by information flow between the amygdala and prefrontal cortex. However, FA provides an overall measure of white matter integrity, which may not essentially be true. Microscopic diffusion values, like RD could better explain local structural changes. Increased RD values are generally interpreted as a sign of myelin sheath disruption and neurodegeneration, however, in our study, based on strict inclusion criteria, this does not hold true, in terms of neurodegeneration.

Increased RD values in the right amygdala in the PC-OlfDys group could indicate changes in myelin structure. Discrimination scores were negatively correlated with RD values in the left amygdala. Potential disruptions in myelin sheath integrity may lead to reduced information flow between key brain regions, mainly amygdala and prefrontal cortex. This could lead to reduced discrimination scores, as discrimination is cognitively demanding^33,34^. The increased RD values in the right amygdala may indicate disruptions in white matter integrity, potentially related to demyelination or axonal damage in pathways associated with olfactory processing. Conversely, the increased FA values in the left amygdala could reflect improved white matter integrity or organization, possibly indicating compensatory mechanisms or adaptations to olfactory deficits.

The association between the duration of olfactory loss and the observed increases in MD and AD values within the left amygdala, as well as with increased MD values in the left posterior piriform cortex, is a point of considerable interest. These regions, recognized as primary olfactory cortex areas, are pivotal in olfactory processing, and any adverse changes within them could indicate persistent OD^35^. MD and AD values, serving as microscopic indicators of local white matter organization, offer valuable insights into the structural integrity of these brain regions. In the context of PC-OlfDys participants, where prolonged olfactory loss was prevalent, this finding is not only corroborated by existing research, including our own^36^, but also highlights the potential significance of these structural alterations. It is captivating to speculate that such changes may serve as fundamental drivers behind the persistent symptoms of PC-OlfDys and may potentially manifest as permanent changes.

The PHQ-8 and GAD-7 questionnaires, commonly used as diagnostic tools to assess depressive and anxiety symptoms, showed divergency in these mental health domains in PC-OlfDys. Although not direct measures of depression and anxiety, it is noteworthy that a negative correlation emerged between these markers and diffusion values in the left amygdala and putamen. Previous research has highlighted reduced functional connectivity between the amygdala and putamen in individuals with major depressive disorder with anxiety component, a prevalent subtype of major depressive disorder^37^. Similarly, changes in the amygdala and putamen were observed in the PC-OlfDys group, with a negative correlation detected between olfactory threshold scores and putamen RD, and between olfactory discrimination and identification scores and amygdala RD. Given that olfactory function, depression, and anxiety are interrelated, with cognitive processes playing an important role, structural changes in the white matter areas surrounding the amygdala are likely to have a considerable influence on both olfactory function and mental health outcomes. This suggests a bidirectional relationship between olfactory function and depression and anxiety, with the absence of feedback, as indicated by a decrease in structural connectivity, potentially perpetuating a vicious cycle. Our results are in line with considerable work in the past suggesting a causal relationship between olfaction, anxiety, and depression^38,39^.

To gain a deeper understanding of the structural consequences of depression, anxiety and OD, a longitudinal study is needed. This will allow to track the progression of the disease and its possible link to depression and anxiety. At this point, a comparison with a post-COVID cohort with depression without OD would also be interesting to see which changes are due to the olfactory deficit and which are due to depression. One of the main limitations of diffusion-based or even functional studies is sample size. Although there is no ideal sample size per se, a sample size of more than 20 participants per group has considerable statistical power for functional or diffusion-based studies^40^.

## Conclusion

In conclusion, this study highlights significant brain changes associated with persistent olfactory dysfunction (OD) following SARS-CoV-2 infection. Using DTI, we identified alterations in key olfactory-related brain regions, particularly in the amygdala, putamen and piriform cortex, where increased fractional anisotropy and radial diffusivity values suggest both enhanced myelination and potential disruptions in white matter integrity. Our findings also suggest that the longer the OD persists after the infection, the greater the DTI changes within critical olfactory circuits. This may have implications for both olfactory function and mental health outcomes. The observed correlations between diffusion metrics, olfactory scores, and depression/anxiety assessments (PHQ-8 and GAD-7) underscore the complex relationship between prolonged OD and its psychological impact.

Our results add to the growing body of evidence that links post COVID-19 condition with cerebral changes, particularly in olfactory processing regions, and suggests that these alterations may be drivers of persistent symptoms. Future longitudinal studies with larger cohorts and potentially further subcohorts are necessary to further elucidate the trajectory of recovery and the potential for structural and functional brain changes to serve as biomarkers for persistent OD and associated mental health conditions.

## Data Availability

Data that support the findings of this study are available from NAPKON but restrictions apply to the availability of these data, which were used under license for the current study, and so are not publicly available. Data are however available from the corresponding authors upon reasonable request and with permission of NAPKON.

## Abbreviations

AD: Axial diffusivity
BET: Brain extraction toolbox
DTI: Diffusion tensor imaging
FA: Fractional anisotropy
FDT: FSL diffusion toolkit
FSL: FMRIB software library
GAD-7: Generalized Anxiety Disorder 7
MD: Mean diffusivity
MoCA: Montreal Cognitive Assessment test
NICE: National Institute for Health and Care Excellence
NAPKON: National Pandemic Cohort Network
OD: Olfactory dysfunction
PC-N: Post-COVID normosmic
PC-OlfDys: Post-COVID olfactory dysfunction
PHQ-8: Eight-item Patient Health Questionnaire-depression
RD: Radial diffusivity
ROI: Region of interest
TBSS: Tract-based spatial statistics
TDI: Threshold-Discrimination-Identification score
VAS: Visual Analogue Scale
WHO: World Health Organization
